# 7.0T ultrahigh-field MRI directly visualized the pedunculopontine nucleus in Parkinson's disease patients

**DOI:** 10.6061/clinics/2019/e573

**Published:** 2019-04-10

**Authors:** Jia-Wei Wang, Fei Cong, Yan Zhuo, Lin Chen, Bo Wang, Yu-Qing Zhang

**Affiliations:** I Department of Neurosurgery, National Cancer Center/National Clinical Research Center for Cancer/Cancer Hospital, Chinese Academy of Medical Sciences and Peking Union Medical College, Chaoyang District, Beijing, 100021, P.R., China; II Department of Functional Neurosurgery, Xuanwu Hospital, Capital Medical University, Xicheng District, Beijing, 100053, P.R., China; III State Key Laboratory of Brain and Cognitive Science, Beijing MR Center for Brain Research, Institute of Biophysics, Chinese Academy of Sciences, Chaoyang District, Beijing, 100101, P.R., China; IV University of Chinese Academy of Sciences, Shijingshan District, Beijing, 100049, P.R., China

**Keywords:** Parkinson's Disease, Pedunculopontine Nucleus, 7.0T MRI, Postural Instability, Gait Disturbance

## Abstract

**OBJECTIVES:**

The pedunculopontine nucleus (PPN) is considered a promising new target for neurostimulation in Parkinson's disease (PD) patients with postural instability and gait disturbance that is refractory to other treatment modalities. However, the PPN is typically difficult to visualize with magnetic resonance imaging (MRI) at clinical field strengths, which greatly limits the PPN as a viable surgical target for deep brain stimulation (DBS). Thus, the aim of this study is to directly visualize the PPN based on 7.0T ultrahigh-field MRI.

**METHODS:**

Five PD patients were enrolled and scanned using the MP2RAGE sequence on a 7.0T ultrahigh-field MRI scanner. Then, the MP2RAGE sequences were imported into a commercially available navigation system. The coordinates of the directly localized PPN poles were recorded in the navigation system relative to the anterior commissure-posterior commissure plane.

**RESULTS:**

Our results indicated that the PPN presented intermediate signal intensity in the 7.0T ultrahigh-field MR images in comparison with the surrounding structure, such as the hypo-intensity of the periaqueductal gray and the hyperintensity of the neighboring white matter tracts, in PD patients. The mean coordinates for the rostral and caudal poles of PPN were 6.50 mm and 7.20 mm lateral, 1.58 mm and 2.21 mm posterior, and 8.89 mm and 13.83 mm relative to the posterior commissure.

**CONCLUSION:**

Our findings provide, for the first time, direct visualization of the PPN using the MP2RAGE sequence on a 7.0T ultrahigh-field MRI, which may improve the accuracy of stereotactic targeting of the PPN and improve the outcomes in patients undergoing DBS.

## INTRODUCTION

As a leading cause of morbidity and death in advanced PD patients, postural instability and gait disturbance (PIGD) has been a great challenge in the clinic because it is commonly refractory to currently available medications 1. Previous studies have demonstrated that the pedunculopontine nucleus (PPN) is an important component of the locomotor region in the brain stem and a key structure in the control of posture and gait 1-3. Thus, to date, there are several completed or ongoing studies on the topic of PPN-DBS for addressing PIGD in PD. However, the enthusiasm for PPN-DBS being potentially capable of treating PIGD has been tempered by mixed and confusing clinical outcomes 1,4-6.

Several critical issues are related to variability in the published data for PIGD treatment 7,8. Chief among these issues is the optimal localization of the PPN. As indicated in the previous literature, the placement of DBS leads during PPN-DBS has been more variable than the placement in other DBS procedures, such as DBS of the subthalamic nucleus (STN) and globus pallidus internus (GPi) 8,9. Compared with the STN or GPi, the PPN is typically difficult to visualize with magnetic resonance imaging (MRI) at clinical field strengths (1.5T and 3.0T) 10. This issue, until resolved, may greatly limit the PPN as a viable surgical target for DBS. With the development of neuroimaging methods, ultrahigh-field (7.0T or higher) MRI seems to provide potential for more accurate targeting of DBS in patients with PD or other movement disorders 10,11.

To the best of our knowledge, there are no reported studies using 7.0T ultrahigh-field MRI for the PPN. Thus, the aim of this study is to use brain images of PD patients based on 7.0T MRI scanners to obtain high quality images of the PPN, which may create a reliable method of PPN localization. Furthermore, the accuracy of PPN-DBS is expected to increase, and the clinical outcomes of PD patients may be improved with the assistance of 7.0T MRI.

## MATERIALS AND METHODS

### Patient characteristics

Five PD patients (1 male and 4 females) with a mean age of 54 years (range 44-64 years) and a mean disease duration of 6 years (range 2-12 years) were recruited for this study. Each enrolled patient completely conformed to the UK PDS Brain Bank diagnostic criteria for idiopathic Parkinson's disease. The institutional review boards of Xuanwu Hospital affiliated with Capital Medical University approved the present study. All enrolled patients provided written informed consent. In addition, the study conformed to the Helsinki Declaration.

### 7.0T ultrahigh-field MRI scanning

The 7.0T MR images were collected at the Siemens Magnetom 7T research system (Siemens, Erlangen Germany) using the MP2RAGE sequence. The scanning parameters were as follows: 0.7 isotropic voxels with 384*384*224 data matrix, TR=4000 ms, TE=3.08 ms, TI1/TI2=900/2750 ms, FA1/FA2=4/5 deg, and bandwidth=240 Hz/Px.

### Stereotactic localization of PPN

The MP2RAGE sequences of every patient were imported into a commercially available navigation system (Stealthstation FrameLink^TM^, Medtronic, Minneapolis, USA) that allowed image viewing in axial, coronal and sagittal planes. Surrounding anatomical landmarks were recognized on the MR images 2, including the anterior commissure (AC), decussating superior cerebellar peduncles (Dec SCP), medial lemniscus (ML), posterior commissure (PC), central tegmental tract (CTT), substantia nigra pars compacta (SNc), cerebral aqueduct (CA) and periaqueductal gray (PAG). We defined the PPN location by these surrounding projection systems that were defined in stereotactic space. In brief, the rostral pole of the PPN can be identified at the mid-inferior collicular level, while the caudal pole lies in the rostral pons with the nucleus spanning approximately 5 mm 9. The coordinates of the PPN poles in relation to the AC-PC plane were recorded in the FrameLink^TM^ system.

## RESULTS

**Figure 1:**
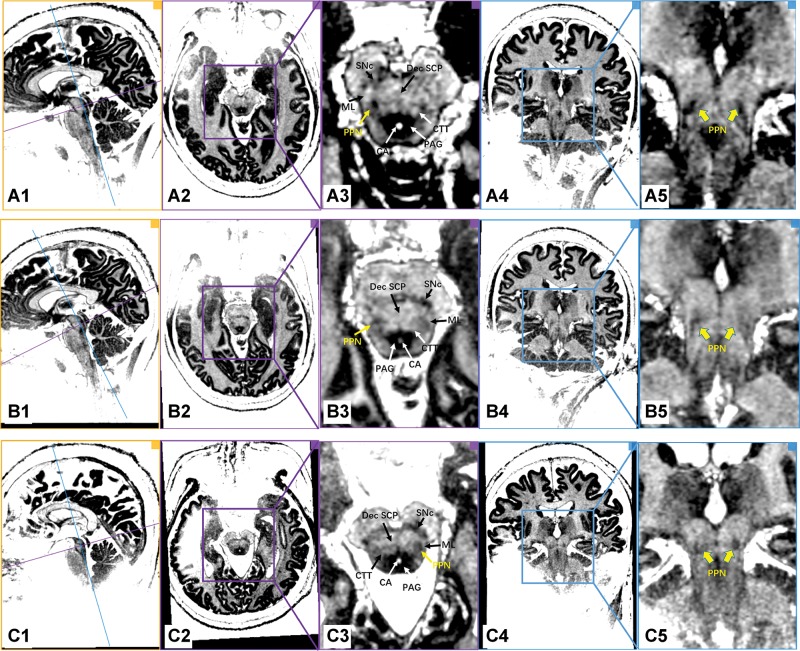
7.0T ultrahigh-field MRI scans using the MP2RAGE sequence in the PD patients (Patients A, B and C). Every row represents the images of the same PD patient. The first column shows the orthographic views for every patient. The two vertically intersecting lines (blue line and purple line) in the first column represent the two planes in other columns (the plane of columns 2/3 with purple border and the plane of columns 4/5 with blue border). The PPN was delineated as a region of intermediate signal intensity when compared to the surrounding structure, such as the hypo-intensity of the PAG and the hyperintensity of the neighboring triad of white matter tracts. Dec SCP: decussating superior cerebellar peduncle, ML: medial lemniscus, CTT: central tegmental tract, SNc: substantia nigra pars compacta, CA: cerebral aqueduct, PAG: periaqueductal gray, PPN: pedunculopontine nucleus.

**Figure 2:**
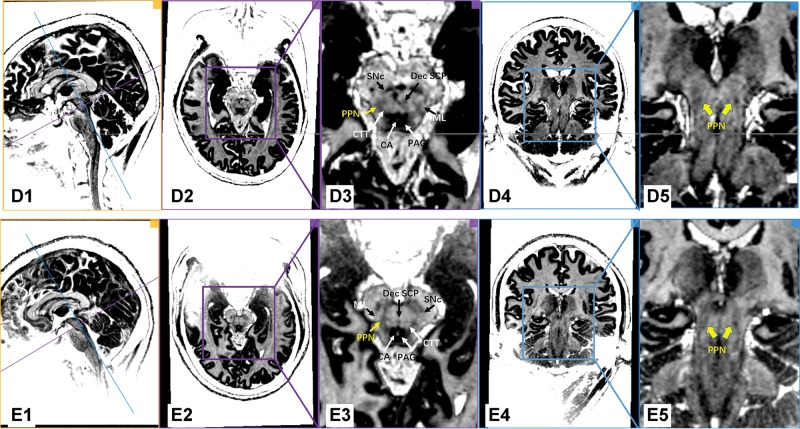
7.0T ultrahigh-field MRI scans using the MP2RAGE sequence in the PD patients (Patients D and E). Every row represents the images of the same PD patient. The first column shows the orthographic views for every patient. The two vertically intersecting lines (blue line and purple line) in the first column represent the two planes in other columns (the plane of columns 2/3 with purple border and the plane of columns 4/5 with blue border). The PPN was delineated as a region of intermediate signal intensity when compared to the surrounding structure, such as the hypo-intensity of the PAG and the hyperintensity of the neighboring triad of white matter tracts. Dec SCP: decussating superior cerebellar peduncle, ML: medial lemniscus, CTT: central tegmental tract, SNc: substantia nigra pars compacta, CA: cerebral aqueduct, PAG: periaqueductal gray, PPN: pedunculopontine nucleus.

Figure 1 shows images of individual PD patients based on the MP2RAGE sequence on the 7.0T ultrahigh-field MRI. With the assistance of excellent gray/white matter differentiation as well as an understanding of local anatomy in the brainstem, the MP2RAGE sequence on the 7.0T ultrahigh-field MRI scans reliably provided a direct visualization of the human PPN (Figure 1 and Figure 2). In our series, white matter tracts appeared hyperintense, while the gray matter or adjacent nuclei within the brainstem were relatively hypo-intense. As indicated in Figure 1, PPN was localized within the gray matter delineated anteromedially by the Dec SCP, anterolaterally by the ML, and posteromedially by the CTT. Moreover, the shape of the rostral PPN in the axial section was commonly irregular, while the shape turned into a narrow boomerang when extending caudally.

The coordinates of the PPN relative to the PC and AC-PC plane in individual PD patients are listed in Table 1. The mean (SD) coordinates for the rostral pole were 6.50 mm (0.46) lateral and 1.58 mm (0.83) posterior to the PC, and 8.89 mm (0.45) caudal to the AC-PC plane. The mean (SD) coordinates of the caudal pole were 7.20 mm (0.36) lateral and 2.21 mm (1.1) posterior to the PC, and 13.83 mm (0.63) caudal to the AC-PC plane. In addition, the rostrocaudal distance of the PPN spanned a mean distance of 4.94 mm (SD: 0.37).

## DISCUSSION

To our knowledge, our findings provide, for the first time, direct visualization of the PPN in PD patients with the use of 7.0T ultrahigh-field MRI. Furthermore, the PPN was delineated as an intermediate signal region in the MP2RAGE sequence compared to the surrounding structures, such as the hypo-intensity of the PAG and the hyperintensity of the neighboring white matter tracts. In addition, we characterized the coordinates of the 7.0T MRI-visualized PPN relative to the traditional AC-PC plane.

It is well known that several methods of verification for specific DBS targets are used during DBS procedures, including neuroimaging, intraoperative microelectrode and local field potential recording, and testing stimulation before permanent implantation. Among these verification methods, anatomical localization based on neuroimaging is accepted as the universal first step of DBS surgery 12. However, variations are commonly experienced with the PPN in comparison with other targets, such as the STN and Gpi. As described in previous studies, T2-weighted and proton density sequences of MR images at common strengths (1.5T and 3.0T), which allows the identification of landmarks circumscribing the PPN region (especially the white matter tracts) to approximate he PPN location, are advocated by most authors 8,9. In comparison with previous methods, our findings may provide a more direct pathway for nucleus imaging. Moreover, when coregistered with postoperative images after DBS implantation, preoperative PPN imaging can provide a spatial location of the DBS lead relative to the target, which may facilitate DBS programming and be useful in explaining abnormal responses.

The AC-PC system and B-F system are the two common references to describe the PPN location 9. According to the study of Zrinzo et al., there was no significant difference between the PPN poles in relation to the B point in comparison with those in relation to the PC based on MRI 9. Thus, in the present study, we used the traditional stereotactic AC-PC system to map the PPN location through the navigation software, which may maximally simulate the DBS procedure in the field. Values for our coordinates of the PPN measured on the 7.0T MR images were 6.50-7.20 mm lateral, 1.58-2.21 mm posterior and 8.89-13.83 mm caudal to the posterior commissure, which is consistent with the reported average coordinates for the target by other authors (6-7.5 mm lateral, 13-15 mm inferior, and 1.5 mm posterior to the PC) 8. These results in turn validated the reliability of the PPN imaging by 7.0T MRI using the MP2RAGE sequence.

In conclusion, 7.0T ultrahigh-field MRI using the MP2RAGE sequence provides direct visualization of the PPN in PD patients, which may improve the accuracy of stereotactic targeting for the PPN and improve the outcomes in patients undergoing DBS.

## AUTHOR CONTRIBUTIONS

Wang JW, Zhang YQ and Cong F performed the MRI scans and wrote the manuscript. Zhuo Y and Chen L analyzed the data. Wang JW and Wang B designed the research.

## Figures and Tables

**Table 1:** Coordinates of the PPN in relation to the PC and AC-PC plane in the five patients.

No.	AC-PC/mm	Sides	Rostral PPN			Caudal PPN		
			Lateral/mm	AP/mm	Vertical/mm	Lateral/mm	AP/mm	Vertical/mm
1	21.89	R	5.89	-1.87	-8.99	7.07	-0.65	-14.89
		L	6.57	-2.59	-9.08	7.58	-1.74	-14.04
2	21.42	R	6.04	-0.90	-8.18	7.25	-1.92	-12.98
		L	6.44	-0.77	-8.96	7.41	-3.20	-13.83
3	22.77	R	6.88	-1.80	-9.55	7.29	-3.73	-14.56
		L	6.99	-1.08	-8.57	7.26	-1.66	-13.65
4	22.95	R	6.25	-0.76	-8.36	6.61	-1.47	-13.07
		L	7.39	-0.85	-8.63	7.79	-1.09	-13.38
5	22.06	R	6.19	-2.12	-9.49	6.69	-2.90	-14.34
		L	6.40	-3.04	-9.07	7.09	-3.75	-13.58

Negative antero-posterior (AP) and rostrocaudal (vertical) values were posterior and caudal to the PC, respectively. R and L represent the right and left patient sides, respectively. AC: anterior commissure, PC: posterior commissure, PPN: pedunculopontine nucleus.
